# Immunoglobulin A vasculitis and pustular psoriasis precipitated by Tawon Liar: a case report

**DOI:** 10.1186/s13256-025-05167-5

**Published:** 2025-05-30

**Authors:** Mengyi Zha, Delaney D. Ding

**Affiliations:** 1 Yakima Valley Farm Workers Clinic, Yakima, WA USA; 2 University of Florida College of Medicine, 1600 SW Archer Road, Gainesville, FL 32610 USA

**Keywords:** Immunoglobulin A (IgA) vasculitis, Pustular psoriasis, Tawon Liar, Adalimumab, Drug-induced vasculitis, Traditional herbal remedies, Food and Drug Administration, Migrant health, Socioeconomics, Medication health fraud, Case report

## Abstract

**Background:**

Unregulated herbal supplements can pose significant health risks due to undisclosed ingredients. Tawon Liar, an Indonesian product marketed as an "all-natural" remedy, claims to alleviate pain and boost immunity but lacks stringent regulatory oversight. We report a unique case of Tawon Liar-induced Immunoglobulin A (IgA) vasculitis and exacerbation of psoriasis, highlighting the potential dangers associated with misadvertised supplements.

**Case presentation:**

A 53-year-old migrant worker from Mexico with a history of psoriasis and ankylosing spondylitis, effectively managed with adalimumab, presented with new-onset rashes on his extremities. Physical examination revealed palpable purpura on the lower legs and erythematous papules and plaques with pustules on the upper extremities. Dermoscopic analysis suggested IgA vasculitis and pustular psoriasis. The patient denied recent infections, new medications, or over-the-counter drug use. However, after thorough questioning, it was revealed that he had been intermittently ingesting Tawon Liar for chronic musculoskeletal pain. The supplement, obtained from a coworker, contained undisclosed ingredients including meloxicam, ketorolac, and dexamethasone. Laboratory tests ruled out renal involvement, and biopsies were not performed due to financial constraints. The patient was advised to discontinue Tawon Liar and was treated with topical corticosteroids, leading to substantial improvement and resolution of symptoms within one week.

**Conclusions:**

This case underscores the potential dangers of herbal supplements containing hidden pharmacologic agents. It highlights the need for clinicians to diligently inquire about supplement use during patient evaluations, especially for vulnerable populations facing language barriers and limited access to healthcare. Public health authorities should enhance efforts to disseminate drug safety information across diverse languages and platforms to mitigate health risks associated with such products.

## Background:

Touted as an “all natural” supplement with medicinal plants and honey extract, Tawon Liar is an Indonesian herbal remedy marketed for pain relief, insomnia, energy enhancement, and cholesterol reduction [1]. Unlike prescription drugs and over-the-counter (OTC) drugs, dietary/herbal supplements like Tawon Liar do not require a formal review for safety by the United States Food and Drug Administration (FDA) before being sold [2], allowing for the distribution of products with undisclosed or inaccurately labeled ingredients, and enabling medication health fraud.

Herbal and dietary supplement use is particularly prevalent among immigrant populations and individuals with limited healthcare access. Studies have shown that foreign-born individuals in the United States often turn to alternative therapies, including herbal supplements, for disease prevention and symptom relief [3]. Additionally, racial and ethnic minorities are more likely to use these remedies but less likely to disclose their usage to healthcare providers, increasing the risk of adverse effects and drug interactions. This disparity underscores the vulnerability of patients like ours, who faced financial and language barriers that influenced his self-medication decisions.

Given the lack of regulatory oversight and the potential for hidden pharmacologic agents in supplements, cases like this highlight the need for clinicians to actively inquire about supplement use, particular in at-risk populations.

## Case presentation

**Fig. 1:**
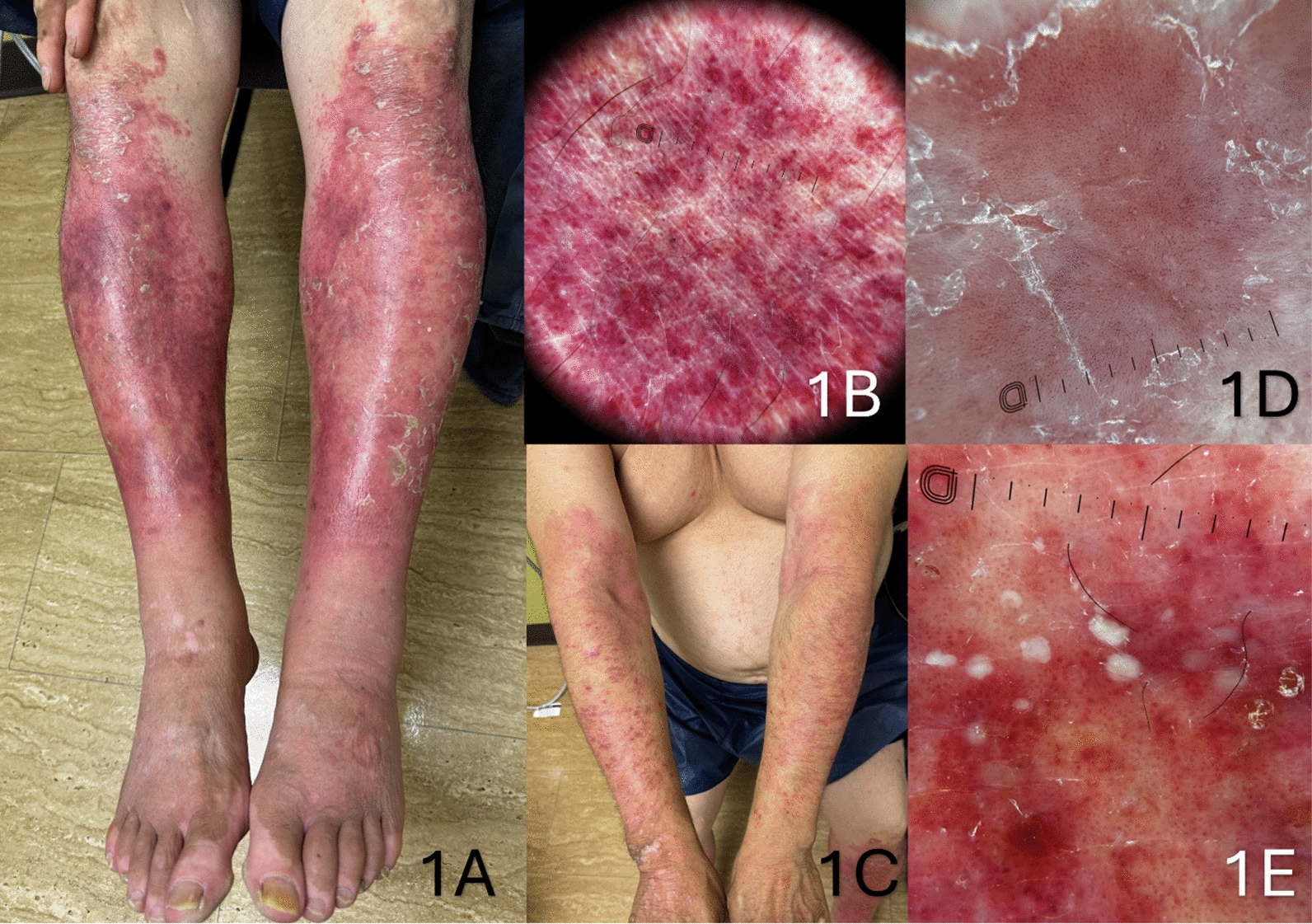
Lower extremities with palpable purpura (**A**), Dermoscopy of lower extremity lesions showing dark pink to purple spots densely dispersed among brown to orange backgrounds (**B**), Upper extremities with scaly erythematous papules and plaques (**C**), Dermoscopy of upper extremity lesions showing dotted blood vessels among pink backgrounds and white scales (**D**), Dermoscopy of pustulosis within the above lesions (**E**)

A 53-year-old Spanish-speaking migrant worker from Mexico with a history of psoriasis and ankylosing spondylitis, previously well-controlled on adalimumab, presented with a new-onset rash of his extremities (*T* = 0 weeks, initial presentation). The patient was accompanied by his wife, who was also Spanish-speaking. The patient denied abdominal pain, vomiting, hematemesis, diarrhea, hematochezia, joint pain, or recent illness. The patient’s rash was distributed across his lower legs and lower arms, sparing the hands and feet. A physical exam revealed palpable purpura on the lower extremities (Fig. 1A). Dermoscopy showed dark pink to purple spots densely dispersed among brown to orange backgrounds (Fig. 1B). In the upper extremities and along the periphery of the lower extremities’ purpuric plaques, there were groups of erythematous papules and plaques (Fig. 1C) with dermoscopic findings of dotted blood vessels among pink backgrounds and white scales (Fig. 1D). Within these papules and plaques, there were clusters of white pustules (Fig. 1E). Exacerbation of psoriasis with pustulosis and Immunoglobulin A (IgA) vasculitis were suspected.

Upon further investigation, the patient reported perfect adherence to adalimumab injections and no recent infections, illnesses, new prescriptions, or recent systemic corticosteroids. These details were confirmed with a thorough chart review. Initially, the patient denied any OTC medication use. However, after much discussion, the patient’s wife admitted that he had been taking a “supplement” containing “bee venom” that his friend at work recommended for muscle pain. Because the patient believed the supplement was made of 100% bee venom, he believed it was an “all-natural” supplement without potential risks. The patient’s occupation demanded physical labor and long hours, inducing chronic musculoskeletal pain and fatigue, so he used $20 to purchase the supplement from his friend, who told him it would help with his muscle pain and give him more energy to stay awake. Each box contained 20 packets of two capsules each, with the patient ingesting the capsules intermittently (that is, one capsule once or twice a day on workdays) for approximately seven months before *T* = 0 weeks (initial presentation). His last dose was five days prior to the onset of the new rash.

**Fig. 2:**
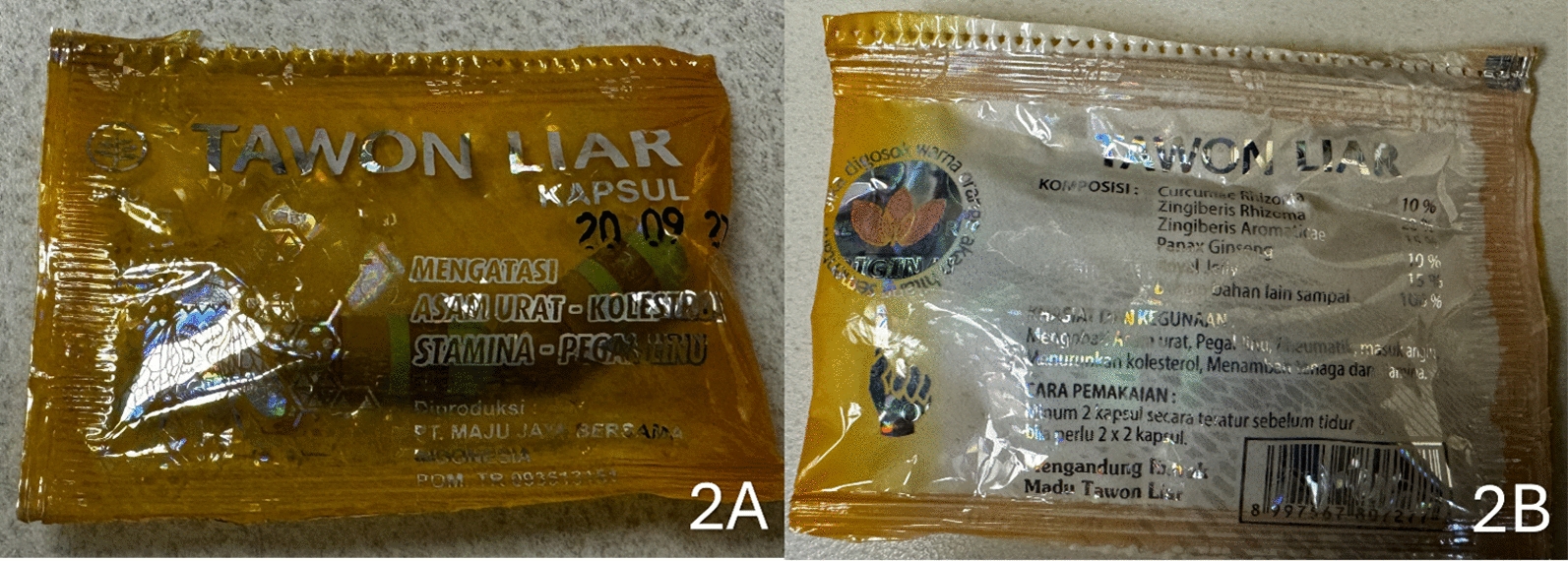
Front packaging of Tawon Liar (**A**), Back packaging of Tawon Liar reporting its ingredients, with ingredient percentages summing to an impossible 155% (**B**)

Neither the patient nor his wife knew the supplement’s name, nor were they aware of the supplement’s online claims. However, the patient's wife was able to retrieve a packet of it from their car. It was Tawon Liar (Fig. 2A), translating to “wild wasp” in Indonesian. The packaging claimed a mathematically illogical distribution of natural ingredients, with ingredient percentages summing to an impossible 155% (Fig. 2B). When sold online, Tawon Liar is touted to benefit digestion, increase stamina, reduce cholesterol, and help people who engage in hard labor or need to stay up late [4]. An internet search revealed a recent FDA warning about hidden ingredients: meloxicam, ketorolac, and dexamethasone [1]. This corroborating information led to the clinical diagnoses of nonsteroidal anti-inflammatory drug (NSAID)-induced IgA vasculitis and systemic corticosteroid-induced exacerbation of psoriasis with pustulosis.

Due to the patient’s lack of health insurance and financial disadvantages, no confirmatory biopsies were performed. A urinalysis was negative for hematuria and a fecal occult blood test was negative for hematochezia. A basic metabolic panel showed normal renal function.

**Fig. 3:**
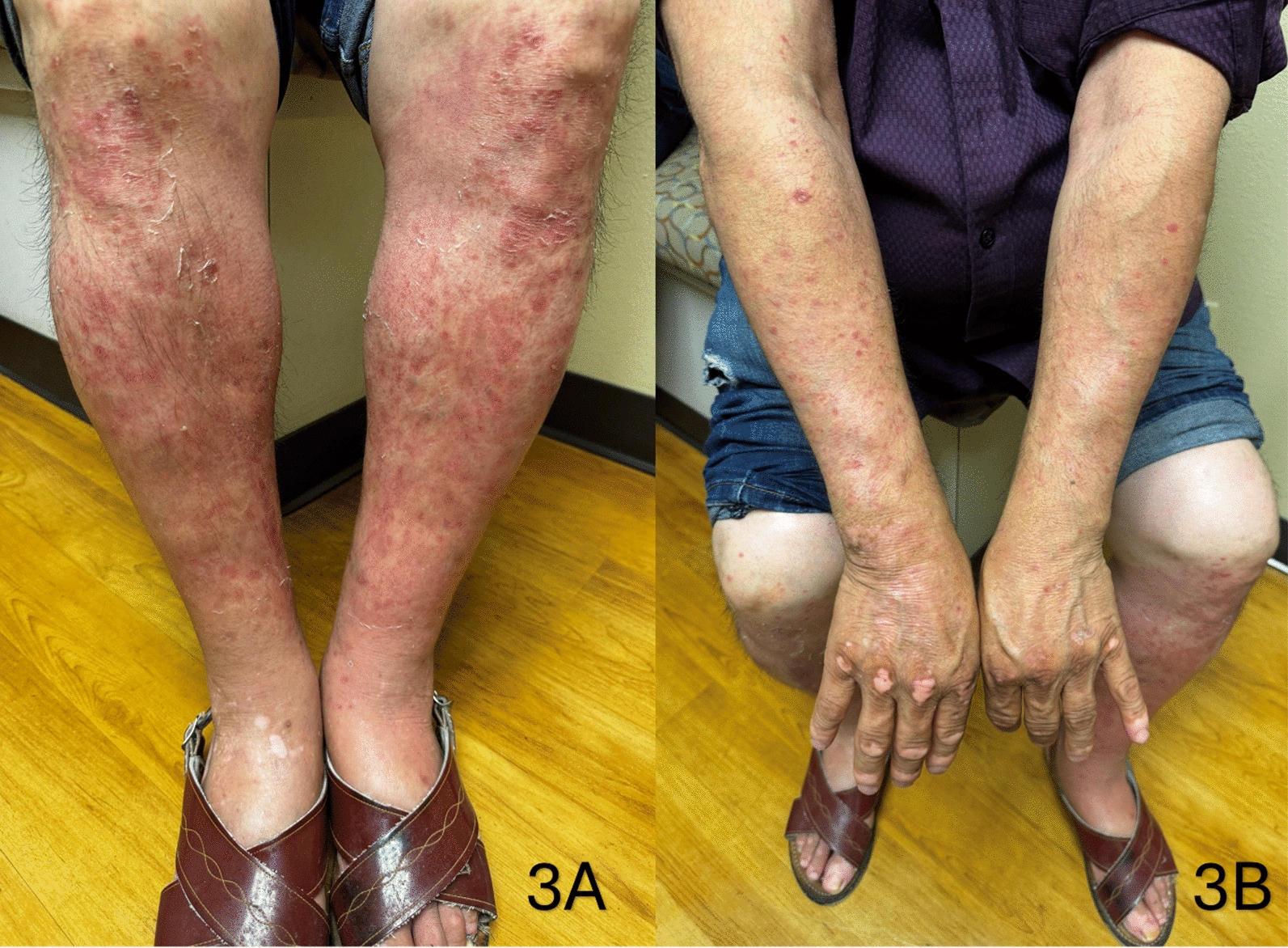
Improvement in lower extremity purpuric lesions one week after stopping Tawon Liar and starting topical steroids (**A**), Improvement in upper extremity lesions one week after stopping Tawon Liar and starting topical steroids (**B**)

The patient was instructed to stop taking Tawon Liar immediately and was treated with 0.1% triamcinolone cream twice a day. On one-week follow-up (*T* = 1 week), he showed significant improvement in both IgA vasculitis and psoriasis, with complete resolution of pustulosis (Fig. 3). On one-month follow up (*T* = 4 weeks), the patient remained clear of the pustular rash or signs of vasculitis without needing topical steroid treatment.

## Discussion and conclusions

IgA vasculitis is an immune-mediated small-vessel vasculitis characterized by palpable purpura, with potential complications like arthritis, gastrointestinal involvement, and renal impairment [5]. Diagnosis is largely based on clinical presentation, supported by laboratory findings and histopathology if needed. Although the most common cause of IgA vasculitis is infections [6], drug-induced IgA vasculitis is an entity demanding increasing attention due to the wide variety of common and accessible medications as culprits. Specifically, NSAIDs and antiplatelet agents have been associated with IgA vasculitis [7].

Recent studies suggest increased IgA plasma cell expression among psoriasis patients [8], which may explain the higher prevalence of IgA nephropathy in this population [9, 10]. This immune dysregulation could contribute to cases involving both IgA vasculitis and psoriasis. In our case, Tawon Liar’s hidden ingredients included meloxicam and ketorolac, both NSAIDs. Ketorolac, in particular, has a boxed warning for bleeding risks, due to its antiplatelet properties. Given its clinically significant propensity for adverse effects, clinical guidelines advise that ketorolac not be used for more than five days. Additionally, concomitant use of ketorolac with other NSAIDs is contraindicated due to the cumulative risk of NSAID-related severe adverse drug reactions [11]. Our patient reported no recent illnesses or vaccinations. Although tumor necrosis factor-alpha inhibitors such as adalimumab have been associated with IgA vasculitis [7], the patient had previously tolerated adalimumab for many years without adverse reactions. Therefore, the most likely culprits for his new-onset IgA vasculitis were meloxicam and ketorolac in Tawon Liar.

The pharmacokinetic properties of these NSAIDs further support this conclusion. Meloxicam has a half-life of approximately 20 h, leading to prolonged systemic effects for several days post-administration [12]. Ketorolac, though having a shorter half-life of approximately 5.6 h, exhibits prolonged pharmacodynamic effects, especially with repeated use [11]. Dexamethasone, with a biological half-life of 36 to 72 h, can exert immunosuppressive effects lasting multiple days after cessation [13]. Given that drug-induced IgA vasculitis typically manifests within 7 to 10 days post-exposure [14, 15], the 5-day interval between the patient's last dose and symptom onset aligns with established timelines for such drug reactions.

Dermoscopy has also proven valuable in diagnosing vasculitis, aiding differentiation between conditions like urticarial vasculitis and other rash presentations [16]. In this case, dermoscopic findings, such as dark pink to purple spots and dotted blood vessels, supported the clinical suspicion of IgA vasculitis. While vasculitis is often readily apparent on dermoscopy due to its purpuric nature, specific patterns provide additional diagnostic insights. Small purpuric patches with blurred borders and red lines on an erythematous background, as described in previous studies, are consistent with findings in our patient [17, 18]. Similarly, pustular psoriasis often presents with red dots and white scales on an erythematous background, distinguishing it from vasculitic lesions [19]. Recognizing these distinctions can assist clinicians in differentiating between IgA vasculitis and pustular psoriasis. Incorporating dermoscopy into routine evaluation of ambiguous rashes can enhance diagnostic accuracy and guide appropriate management by distinguishing between these overlapping conditions.

Abrupt withdrawal of systemic steroids can lead to the pustular transformation of psoriasis. Systemic steroids can precipitate pustular psoriasis by initially suppressing the body’s immune response and subsequently triggering a rebound response involving upregulated cytokine proliferation and accumulation of neutrophils in the epidermis [20]. NSAIDS, such as meloxicam and ketorolac, inhibit prostaglandin synthesis by blocking cyclooxygenase (COX)-1 and COX-2, which may further exacerbate inflammatory pathways in psoriasis. Prostaglandins, particularly PGE2, play a regulatory role in inflammation, and their inhibition has been linked to increased IL-23 and IL-17 signaling, which are key cytokines in psoriasis pathogenesis [21]. The patient reported intermittent self-medication with Tawon Liar, which contains dexamethasone, likely resulting in recurrent steroidal withdrawal, contributing to pustular transformation of his previously well-controlled psoriasis.

Upon initial presentation at *T* = 0 weeks, the patient was instructed to stop taking Tawon Liar immediately and was treated with 0.1% triamcinolone cream twice a day. On one-week follow-up (*T* = 1 week), he showed significant improvement in both IgA vasculitis and psoriasis, with resolution of pustulosis (Fig. 3A, B). Given the pharmacokinetics of the implicated medications, including dexamethasone’s prolonged immunosuppressive effects and NSAIDs’ known potential to induce IgA vasculitis, the timing of symptom resolution closely followed the discontinuation of Tawon Liar. While the initiation of topical corticosteroids may have contributed to the improvement in psoriasis, the rapid resolution of the vasculitic lesions provides supportive evidence for a potential causal link between Tawon Liar and the patient’s presentation. On one-month follow-up (*T* = 4 weeks), the patient remained clear of the pustular rash and vasculitic lesions without the need for continued topical steroid therapy, further supporting the primary role of supplement discontinuation in symptom resolution.

Although the presenting evidence strongly supports Tawon Liar as the causative agent, we acknowledge the limitations due to the lack of a biopsy and the need to consider potential alternative explanations. For example, it is possible that the patient experienced an idiopathic flare of psoriasis unrelated to the supplement, coinciding with the development of IgA vasculitis due to another unidentified trigger. However, this explanation is less likely given the patient’s long history of stable psoriasis on adalimumab and the absence of recent infections or medication changes, apart from Tawon Liar use. Additionally, tumor necrosis factor-alpha inhibitors such as adalimumab have been associated with IgA vasculitis, but the patient had tolerated this therapy for years without prior adverse effects. Acute generalized exanthematous pustulosis (AGEP) is another possibility, but an unlikely diagnosis in this case, as it is typically an acute febrile pustular eruption triggered by medication in about 90% of cases, usually manifesting within 48 h to two weeks of drug exposure rather than after months of intermittent use [22]. Additionally, AGEP does not typically present with vasculitis, further reducing its likelihood in our patient. Similarly, while tumor necrosis factor-alpha inhibitors such as adalimumab have been associated with pustular eruptions as paradoxical reactions, these cases are rare and primarily reported in case studies [23]. Moreover, our patient had been well-controlled on adalimumab for years, and his symptoms resolved without discontinuation of the drug, with no recurrence at one-month follow-up. While we ultimately cannot definitively exclude other contributing factors, the temporal relationship between supplement discontinuation and symptom resolution remains the most compelling explanation for this case.

Tawon Liar not only contains two NSAIDs, one of which is a high-risk medication, but also provides no warning regarding the appropriate duration of usage. It also contains systemic steroids, the chronic use of which can have deleterious effects. Furthermore, pustular psoriasis, especially if generalized, is associated with systemic complications. The potential harms associated with Tawon Liar are much more than skin-deep. The dishonest disclosure of ingredients in this drug is irresponsible, harmful, and potentially life-threatening.

Undisclosed pharmaceutical ingredients in herbal supplements have been implicated in a growing number of adverse drug reactions worldwide. Studies have documented the presence of corticosteroids, NSAIDs, and even prescription medications in unregulated supplements, with risks of significant adverse clinical manifestations [24], which can result in delayed diagnosis and mismanagement of patients. Our case of Tawon Liar further underscores the risks posed by such products, emphasizing the importance of heightened vigilance in identifying supplement-related adverse events.

Addressing these risks requires coordinated efforts among clinicians, regulatory bodies, and public health organizations. Clinicians should prioritize thorough medication reconciliation, including routine screening for supplement use, especially in at-risk populations. Increased regulatory oversight is essential to enforce stricter labeling requirements and implement more rigorous safety evaluations for herbal supplements. Public health initiatives should also focus on education, equipping patients with the knowledge to recognize the risks of unregulated supplements and the importance of disclosing their use to healthcare providers. Additionally, surveillance systems must be improved to track and respond to adverse events associated with these products more effectively. Strengthening these measures can help mitigate the risks posed by mislabeled and adulterated supplements, ultimately improving patient safety.

Special measures should also be taken for marginalized communities. While the prevalence of herbal/dietary supplement usage is high among racial/ethnic minorities [3], compared to non-Hispanic White Americans, Hispanic Americans are less likely to disclose their use [25]. Since patients with language barriers are also less likely to receive adequate pain control [26], it is unsurprising that patients such as ours may resort to self-medication. The patient’s immigration status, lack of health insurance, language and cultural barriers, lack of medical literacy, inadequately treated pain, and occupational risks all contribute to his vulnerability to medication health fraud. Through this patient’s mysterious medical case, we caught a glimpse of the racial, cultural, and socioeconomic barriers that should not be overlooked in healthcare.

When caring for members of marginalized communities such as migrant workers, clinicians should pay special attention to OTC dietary/herbal supplements during medication reconciliation efforts. Some best practices include asking open-ended but specific questions in a culturally sensitive way (such as “Do you take any vitamins?”), engaging family or caregivers, cultivating curiosity rather than judgment among healthcare team members, and whenever possible, examining the medications themselves and their compositions in person. This should be followed by efforts to verify the sources of these medications, self-educate, and provide clear communication to patients and families. Additionally, this case provides an opportunity for involved clinicians to examine their intrinsic biases in inquiring about and managing pain for patients with language and cultural barriers. Furthermore, public health authorities should make drug warnings readily accessible to individuals of all backgrounds by disseminating messages in a broad array of languages and mediums catered to vulnerable patient populations.

## Data Availability

Not applicable.

## Abbreviations

FDA: Food and Drug Administration
OTC: Over-the-counter
IgA: Immunoglobulin A
NSAID: Nonsteroidal anti-inflammatory drug
